# Supplementary material for: Unconventional self-similar Hofstadter superconductivity from repulsive interactions
Source: Nat Commun. 2022 Dec 16;13:7785. doi: 10.1038/s41467-022-35316-z (PMC9758186; doi:10.1038/s41467-022-35316-z)
Supplement: Supplementary file 1 — Supplementary Information [file 41467_2022_35316_MOESM1_ESM.pdf]

Supplementary Material for:
Unconventional Self-Similar Hofstadter Superconductivity from Repulsive Interactions

Supplementary Section I: Details of the RG Calculation

Here we present some details of the diagrammatic RG calculations. Fig. 1 shows all the relevant Feynman diagrams representing the projected interactions in Eqs. (4-5). In addition to the diagrams in Fig. 1(b) in the main text, there are additional processes $g_{2'}$, $g_{3'}$ and $g_{4'}$ related by commutation relations to the g_2 and g_3 processes and by hermiticity to the g_4 process, respectively. As mentioned in the main text, hermiticity and commutation relations, along with MTG symmetries, impose several relations on the projected interactions:

$$\begin{aligned} g_{mn}^{(\ell)1} &= g_{nm}^{(\ell),1*} = g_{-\ell-m,-\ell-n}^{(\ell)1} \\ g_{mn}^{(\ell)1'} &= g_{nm}^{(\ell),1'*} = g_{-1-\ell-m,-1-\ell-n}^{(\ell)1'} \\ g_{mn}^{(\ell)2} &= g_{nm}^{(\ell),2*} = g_{-\ell-m,-\ell-n}^{(\ell)2'} \\ g_{mn}^{(\ell)3} &= g_{-\ell-n,-\ell-m}^{(\ell),3*} = g_{-\ell-m,-\ell-n}^{(\ell)3'} \\ g_{mn}^{(\ell)4} &= g_{-\ell-m,-\ell-n-1}^{(\ell),4} = g_{nm}^{(\ell)4'*} \end{aligned} \tag{S1}$$

These are in addition to the MTG-imposed relation $g_{mn}^{(\ell),j} = g_{m-1,n-1}^{(\ell+2),j}$; note the TRS is broken for $q > 2$, which means that some coupling constants may be complex. There is a total of $2q(q+1)+1$ complex coupling constants for odd q and $2q(q+2)$ for even q; counting only independent *real* parameters, this amounts to $4q^2+2$ parameters for odd q and $4q^2+2q$ for even q.

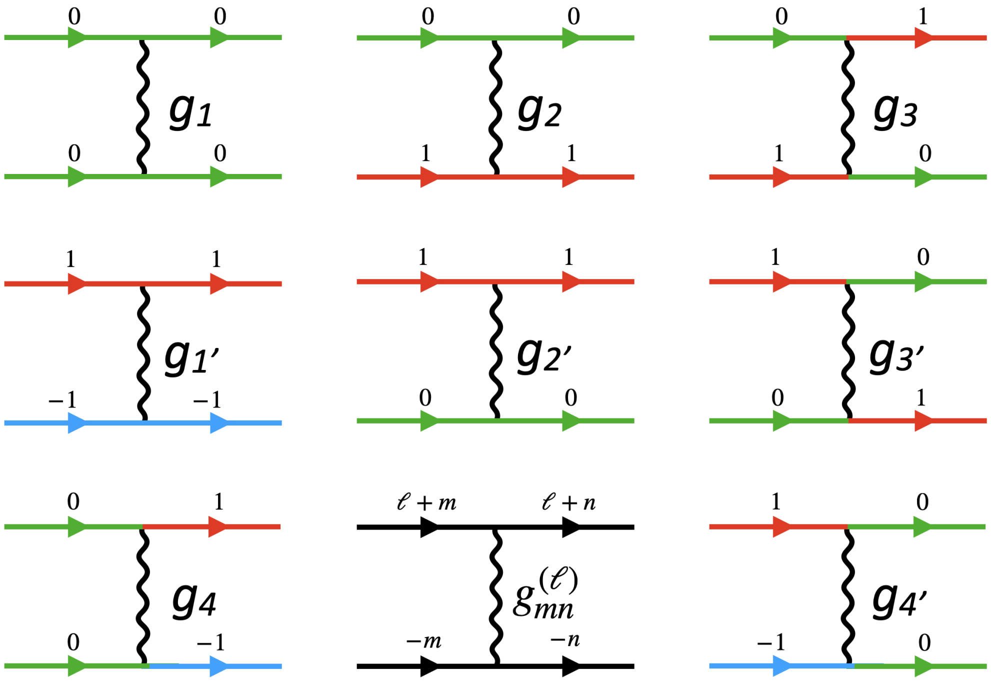

Supplementary Fig. 1. Feynman diagrams representing the interaction processes in Eq. (4) and (5). The colored diagrams show the intrapatch processes g_j (green lines correspond to $v = 0$, red to $v = 1$ and blue to $v = -1$). The black diagram shows the interpatch processes with ℓ labeling the total momentum of the incoming and outgoing pairs (which is conserved), while m and n label relative momenta of incoming and outgoing pairs respectively (so the momentum transfer is labeled by $m - n$). Color online.

In addition, recall that we introduced a redundant VHS index $v = -1$, with the relation $\mathbf{K}_{\ell,-1} = \mathbf{K}_{\ell-1,1}$. This is done in order to avoid diagrams such as the g_4 process in the top left in Fig. 2 in which neither the sum of rMBZ

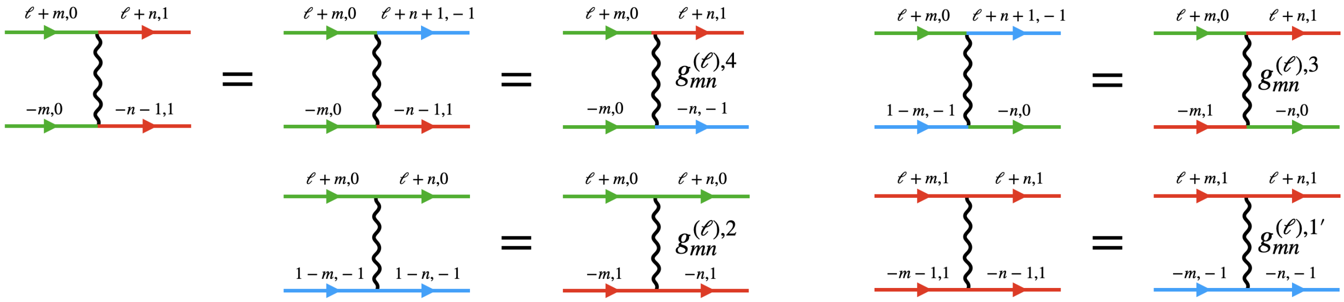

Supplementary Fig. 2. Redundant diagrams introduced to separately conserve patch and intrapatch indices. In all RG equations, the diagrams on the left are replaced with the diagrams on the right whenever they occur to avoid introducing additional redundant coupling constants. Green, red, and blue colors correspond to VHS indices $\mathrm{v} = 0, 1, -1$ respectively (color online).

magnetic flavor indices $\ell, m, n = 0, \ldots, q-1$ nor the sum of the VHS indices v, u, w is equal for incoming and outgoing pairs. This allows us to treat both rMBZ and VHS indices as conserved quantities, but introduces some redundant diagrams also shown in Fig. 2. Such redundant diagrams are to be replaced with diagrams in Fig. 1(b) whenever they appear in the diagrammatic expansion.

In order to compute the RG equations, we need the particle-particle bubble, which also serves as the RG time $t = \Pi_{pp}^{(0)}$ with

$$\Pi_{pp}^{(\mathrm{v})} = -iT \sum_{\omega} \int G_{n,\mathrm{v}}(i\omega, \mathbf{p}) G_{\ell-n,\mathrm{v}}(-i\omega, -\mathbf{p}) \frac{d^2p}{(2\pi)^2} = \nu_0 \log^2 \frac{\Lambda}{E} \tag{S2}$$

The particle-hole bubble is similarly defined as

$$\Pi_{ph}^{(\mathrm{v})} = iT \sum_{\omega} \int G_{n,\mathrm{v}}(i\omega, \mathbf{p}) G_{\ell+n,\mathrm{v}+1}(i\omega, \mathbf{p}) \frac{d^2p}{(2\pi)^2} = \nu_0 \log^2 \frac{\Lambda}{E} \tag{S3}$$

(note that neither bubble depends on the choice of n and ℓ by MTG symmetry; further note that the inter-VHS particle-particle and intra-VHS particle-hole bubbles vanish). Here

$$G_{n,\mathrm{v}}(i\omega, \mathbf{p}) = \frac{1}{i\omega - \varepsilon_{n,\mathrm{v}}(\mathbf{p})} \tag{S4}$$

is the Green's function for the nth magnetic flavor and v^{th} VHS, and E is the energy scale down to which the high energy modes have been integrated out to. The dispersion expanded around the VHS points is $\varepsilon_{\ell,\mathrm{v}}(\mathbf{p}) \approx \pm(-1)^{\mathrm{v}} \frac{p_x^2 - p_y^2}{2m} - \mu$ (the $\pm$ depends on which Hofstadter band the chemical potential is in, but does not alter the calculations). The extra logarithm comes from the diverging DOS at the VHSs. We then define $d_{pp}^{(\mathrm{v})} = \frac{d\Pi_{pp}^{(\mathrm{v})}}{d\Pi_{pp}^{(0)}} \approx \frac{\Pi_{pp}^{(\mathrm{v})}}{\Pi_{pp}^{(0)}}$ and $d_{ph}^{(\mathrm{v})} = \frac{d\Pi_{ph}^{(\mathrm{v})}}{d\Pi_{pp}^{(0)}} \approx \frac{\Pi_{ph}^{(\mathrm{v})}}{\Pi_{pp}^{(0)}}$. Note that due to the $\hat{C}_4$ symmetry $d_{pp}^{(0)} = d_{pp}^{(1)} = 1$ and $d_{ph}^{(0)} = d_{ph}^{(1)}$. We thus drop the superscripts.

To obtain the standard 1 loop RG flow equation, we use the diagrams in Fig. 4, plugging in the magnetic flavor indices from Fig. 3 and using the relations in Fig. 2 where necessary. This yields the RG flow equations shown in Eq. (7) in the main text.

Vertices

The vertices introduced in Eq. (8) also satisfy several symmetry relations. In the spin-singlet pairing channel, anti-commutation relations imply the particle-hole symmetry (PHS) relation

$$\Delta_{m;\mathrm{v}}^{(\ell)} = \Delta_{\ell-m;-\mathrm{v}}^{(\ell)} \tag{S5}$$

In the density wave channels, hermiticity implies $\rho_{m;0}^{[\ell]} = \rho_{m+\ell;1}^{[1-\ell]*}$, and similarly $M_{m;0}^{[\ell,j]} = M_{m+\ell;1}^{[1-\ell,j]*}$. Here we use the notation $M_{m;\mathrm{v}}^{[\ell,j]}$ to denote the jth component of $\mathbf{M}_{m;\mathrm{v}}^{[\ell,j]}$ (we can include CDW as a special case with $j = 0$, $\rho^{[\ell]} = M^{[\ell,0]}$).

The action of the MTG symmetries on the vertices is as follows:

$$\Delta^{(\ell)}_{m;\mathrm{v}} \xrightarrow{\hat{T}_x} \Delta^{(\ell-2)}_{m+1;\mathrm{v}} \tag{S6}$$
$$\Delta^{(\ell)}_{m;\mathrm{v}} \xrightarrow{\hat{T}_y} \omega_q^{p\ell}\Delta^{(\ell)}_{m;\mathrm{v}}$$
$$M^{[\ell,j]}_{m;\mathrm{v}} \xrightarrow{\hat{T}_x} \omega_q^{-1/2}M^{[\ell,j]}_{m-1;\mathrm{v}}$$
$$M^{[\ell,j]}_{m;\mathrm{v}} \xrightarrow{\hat{T}_y} \omega_q^{p\ell-1/2}M^{[\ell,j]}_{m;\mathrm{v}}$$

(CDW included as $j = 0$). Observe that SC is a charge $2e$ order while CDW and SDW is a charge $0e$ order (since the latter orders don't break the $U(1)$ symmetry). The irreps for SC order were classified in [S1]: there is a single q dimensional irrep for odd q and four $q/2$ dimensional irreps for even q. In contrast, the C/SDW orders can be seen to transform according to 1D irreps of usual order q translations forming the $\mathbb{Z}_q^2$ group (labeled by two numbers, the eigenvalues of the order under $\hat{T}_x$ and $\hat{T}_y$). The 1 loop RG flow equations for the vertices given in Eq. (S7) is obtained from the diagrams shown in Fig. 5 and 6. This yields the RG vertex flow equations

$$\dot{\Delta}^{(\ell)}_{m;0} = -g^{(\ell)1}_{nm}\Delta^{(\ell)}_{n;0} - g^{(\ell)4*}_{mn}\Delta^{(\ell)}_{n;1} \tag{S7}$$

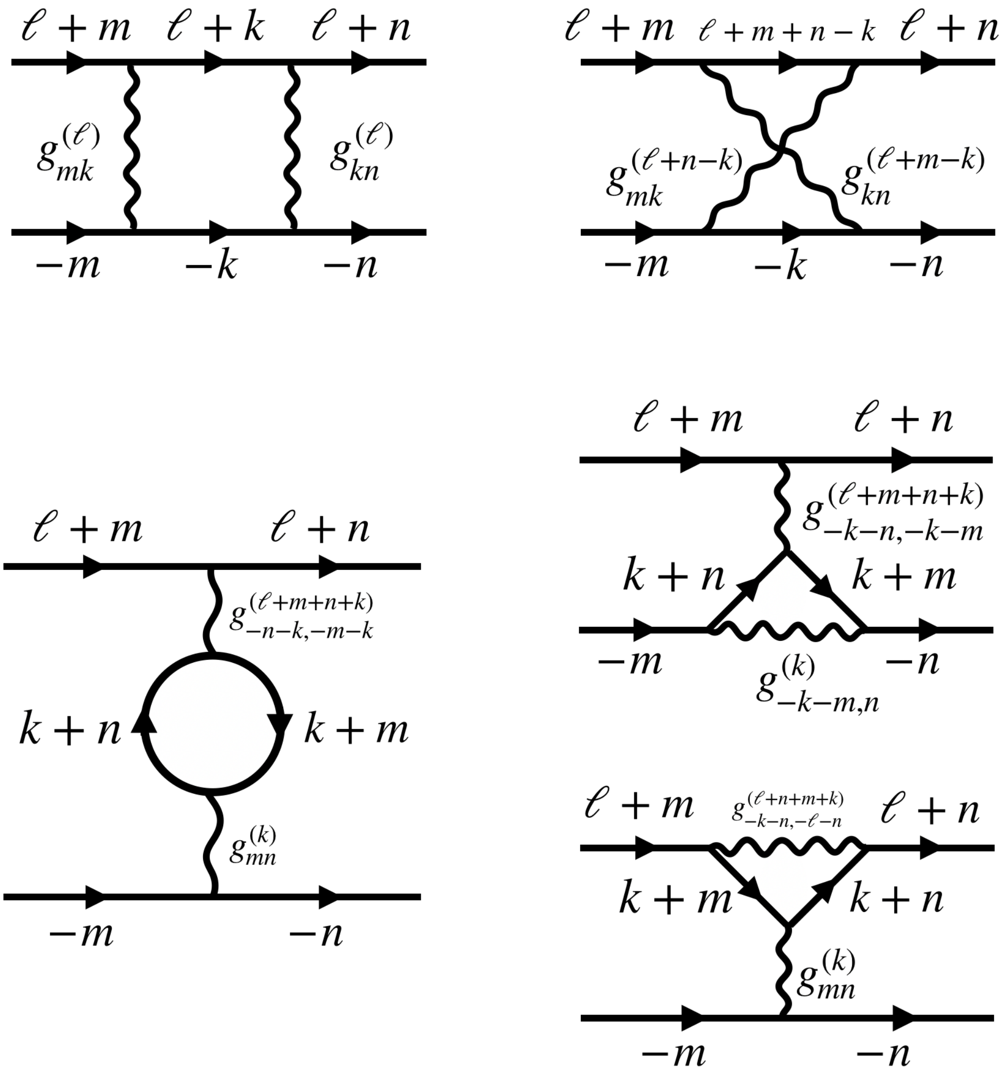

Supplementary Fig. 3. The 1 loop Feynman diagrams renormalizing the interactions for magnetic flavor indices. Wavy lines indicate spin is conserved where they meet the fermion lines.

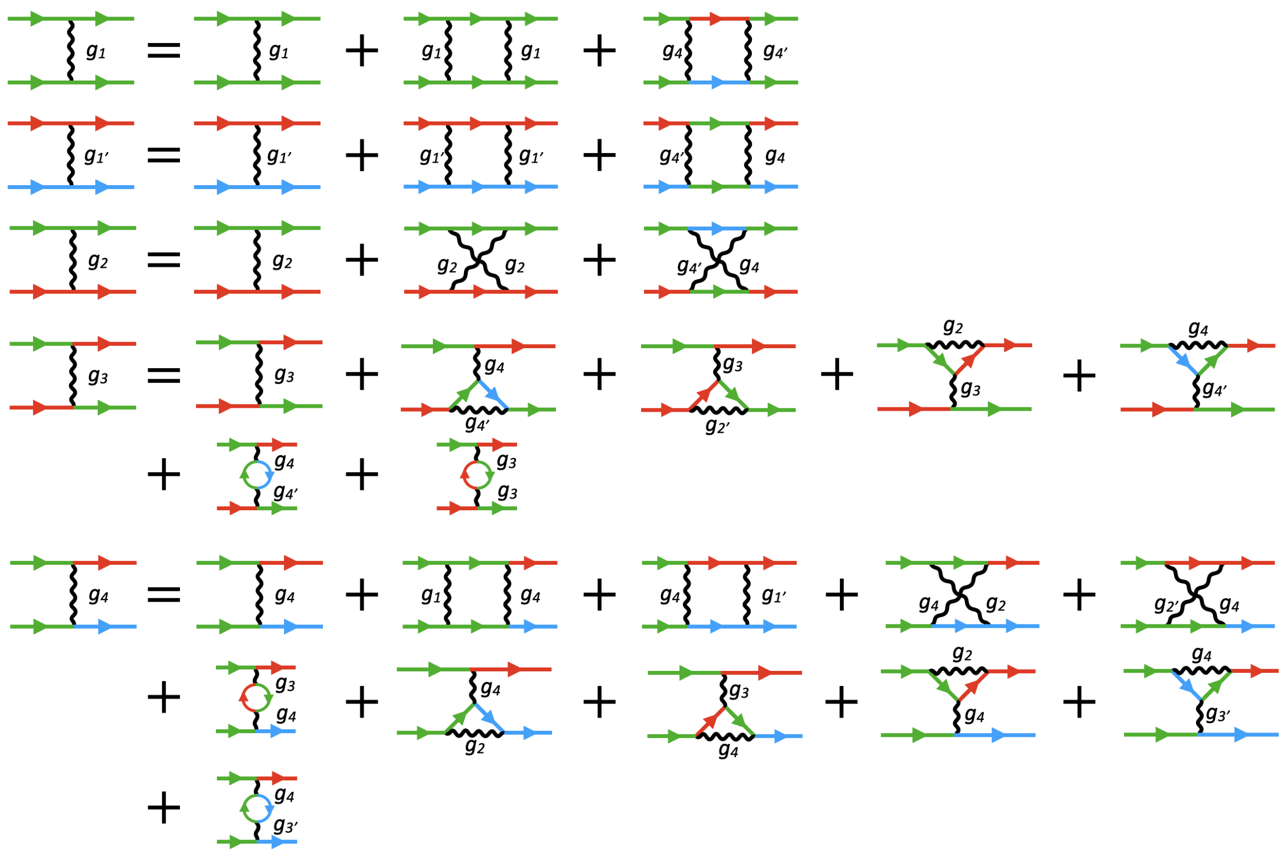

Supplementary Fig. 4. The 1 loop Feynman diagrams RG equations for g_j channels. The magnetic flavor indices ℓ, m, n are to be inserted from Fig. 3

$$
\begin{aligned}
\dot{\Delta}^{(\ell)}_{m;1} &= -g^{(\ell)4}_{nm}\Delta^{(\ell)}_{n;0} - g^{(\ell)1'}_{nm}\Delta^{(\ell)}_{n;1} \\
\dot{\rho}^{[\ell]}_{m;0} &= d_{ph}\left(g^{(\ell+m-n)2}_{n-m,0} - 2g^{(\ell-m+n)3}_{0,-\ell}\right)\rho^{[\ell]}_{n;0} + d_{ph}\left(g^{(\ell+m-n)4*}_{0,n-m-1} - 2g^{(\ell+m-n)4*}_{0,-\ell}\right)\rho^{[\ell]}_{n;1} \\
\dot{\rho}^{[\ell]}_{m;1} &= d_{ph}\left(g^{(\ell+m-n)4}_{n-m,-1} - 2g^{(\ell-m+n)4}_{0,-\ell}\right)\rho^{[\ell]}_{n;0} + d_{ph}\left(g^{(\ell+m-n-1)2}_{1-\ell,1-\ell+n-m} - 2g^{(\ell+m-n-1)3}_{1-\ell,0}\right)\rho^{[\ell]}_{n;1} \\
\dot{M}^{[\ell]}_{m;0} &= d_{ph}\left(g^{(\ell+m-n)2}_{n-m,0}M^{[\ell]}_{n;0} + g^{(\ell+m-n)4*}_{0,n-m-1}M^{[\ell]}_{n;1}\right) \\
\dot{M}^{[\ell]}_{m;1} &= d_{ph}\left(g^{(\ell+m-n)4}_{n-m,-1}M^{[\ell]}_{n;0} + g^{(\ell+m-n-1)2}_{1-\ell,1-\ell+n-m}M^{[\ell]}_{n;1}\right)
\end{aligned}
$$

Note that the MTG symmetries imply that the flow for $\Delta^{(\ell)}_{m;\mathrm{v}}$ is the same for all ℓ for odd q (i.e. each ℓ is a degenerate channel, as a consequence of the irrep being q dimensional), while for even q the channels are only degenerate for ℓ of the same parity and decouple into $\Delta^{(\ell,\pm)}_{m;\mathrm{v}} = \Delta^{(\ell)}_{m;\mathrm{v}} \pm \Delta^{(\ell)}_{m+q/2;\mathrm{v}}$ that flow independently for $+$ and $-$ (a total of four pairing channels, as a result of there being four $q/2$-dimensional irreps in this case). The C/SDW channels, on the other hand, split into q independent equations for each ℓ. Moreover, note that the C/SDW equations are all of the form $\dot{\rho}_m = G_{m-n}\rho_n$ in the magnetic flavor indices, which is a convolution. The C/SDW channels therefore further decouple into their discrete Fourier components

$$
\tilde{M}^{[\ell,j]}_{k;\mathrm{v}} = \sum_m \omega_q^{mk} M^{[\ell,j]}_{m;\mathrm{v}} \tag{S8}
$$

which flow as

$$
\begin{aligned}
\dot{\tilde{\rho}}^{[\ell]}_{k;0} &= \tilde{g}^{(\ell)\rho}_{k;00}\tilde{\rho}^{[\ell]}_{k;0} + \tilde{g}^{(\ell)\rho}_{k;01}\tilde{\rho}^{[\ell]}_{k;1} \\
\dot{\tilde{\rho}}^{[\ell]}_{k;1} &= \tilde{g}^{(\ell)\rho}_{k;10}\tilde{\rho}^{[\ell]}_{k;0} + \tilde{g}^{(\ell)\rho}_{k;11}\tilde{\rho}^{[\ell]}_{k;1} \\
\dot{\tilde{M}}^{[\ell]}_{m;0} &= \tilde{g}^{(\ell)M}_{k;00}\tilde{M}^{[\ell]}_{k;0} + \tilde{g}^{(\ell)M}_{k;01}\tilde{M}^{[\ell]}_{k;1}
\end{aligned} \tag{S9}
$$

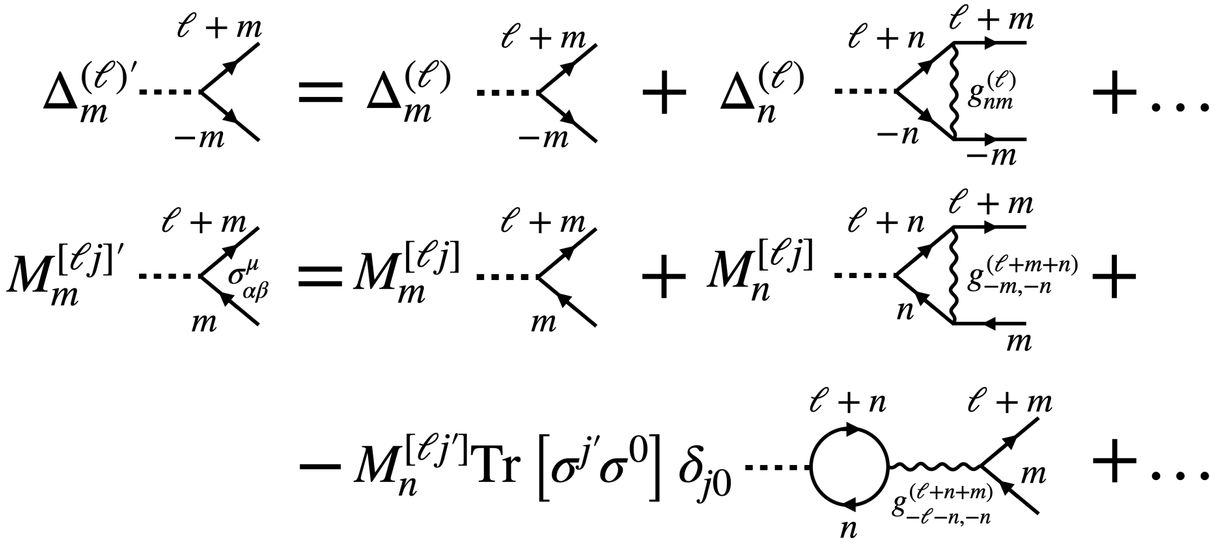

Supplementary Fig. 5. The 1 loop Feynman diagrams renormalizing the vertices for magnetic flavor indices. The last diagram includes a trace over the spin indices and comes with an additional minus sign.

$$\dot{\tilde{M}}_{m;1}^{[\ell]} = \tilde{g}_{k;10}^{(\ell)M}\tilde{M}_{k;0}^{[\ell]} + \tilde{g}_{k;11}^{(\ell)M}\tilde{M}_{k;1}^{[\ell]}$$

where

$$\begin{aligned}
\tilde{g}_{k;00}^{(\ell)\rho} &= d_{ph}\sum_m \omega_q^{mk}\left\{g_{-m,0}^{(\ell+m)2} - 2g_{0,-\ell}^{(\ell-m)3}\right\} \\
\tilde{g}_{k;01}^{(\ell)\rho} &= d_{ph}\sum_m \omega_q^{mk}\left\{g_{-\ell-m,-\ell}^{(\ell+m)4*} - 2g_{0,-\ell}^{(\ell+m)4*}\right\} \\
\tilde{g}_{k;10}^{(\ell)\rho} &= d_{ph}\sum_m \omega_q^{mk}\left\{g_{-\ell,-\ell-m}^{(\ell+m)4} - 2g_{0,-\ell}^{(\ell-m)4}\right\} = \tilde{g}_{k;01}^{(\ell)\rho*} \\
\tilde{g}_{k;11}^{(\ell)\rho} &= d_{ph}\sum_m \omega_q^{mk}\left\{g_{1-\ell,1-\ell-m}^{(\ell+m-1)2} - 2g_{1-\ell,0}^{(\ell+m-1),3}\right\} \\
\tilde{g}_{k;00}^{(\ell)M} &= d_{ph}\sum_m \omega_q^{mk} g_{-m,0}^{(\ell+m)2} \\
\tilde{g}_{k;01}^{(\ell)M} &= d_{ph}\sum_m \omega_q^{mk} g_{-\ell-m,-\ell}^{(\ell+m)4*} \\
\tilde{g}_{k;10}^{(\ell)M} &= d_{ph}\sum_m \omega_q^{mk} g_{-\ell,-\ell-m}^{(\ell+m)4} = \tilde{g}_{k;01}^{(\ell)M*} \\
\tilde{g}_{k;11}^{(\ell)M} &= d_{ph}\sum_m \omega_q^{mk} g_{1-\ell,1-\ell-m}^{(\ell+m-1)2}
\end{aligned} \tag{S10}$$

Note that C/SDW channels thus decouple into q^2 2×2 systems of equations which can be easily further decoupled, all as a consequence of the MTG symmetries (which in this case act simply as regular translations). The corresponding eigen-channels flow as

$$\begin{aligned}
\dot{\tilde{\rho}}_{k;\pm}^{[\ell]} &= \gamma_{k;\pm}^{[\ell]\rho}\tilde{\rho}_{k;\pm}^{[\ell]} \\
\dot{\tilde{M}}_{k;\pm}^{[\ell]} &= \gamma_{k;\pm}^{[\ell]M}\tilde{M}_{k;\pm}^{[\ell]}
\end{aligned} \tag{S11}$$

where

$$\tilde{\rho}_{k;\pm}^{[\ell]} = \left(\tilde{g}_{k;00}^{(\ell)\rho} - \tilde{g}_{k;11}^{(\ell)\rho} \pm \sqrt{(\tilde{g}_{k;00}^{(\ell)\rho} - \tilde{g}_{k;11}^{(\ell)\rho})^2 + 4\left|\tilde{g}_{k;10}^{(\ell)\rho}\right|^2}\right)\tilde{\rho}_{k;0}^{[\ell]} + 2\tilde{g}_{k;10}^{(\ell)\rho}\tilde{\rho}_{k;1}^{[\ell]} \tag{S12}$$

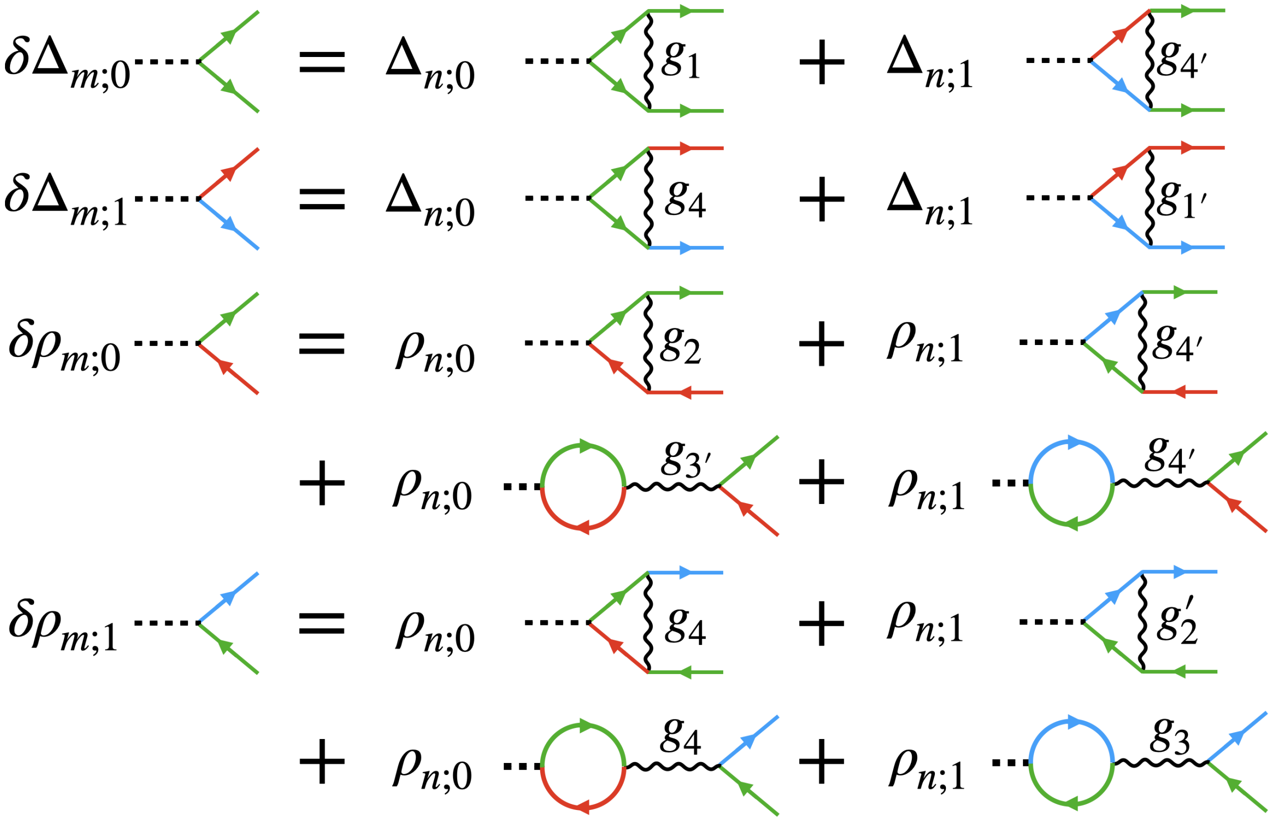

Supplementary Fig. 6. Same as Fig. 3 in the main text. The 1 loop Feynman diagrams renormalizing the SC and CDW vertices for VHS indices (green for v = 0, red/blue for v = ±1 respectively). SDW diagrams are the same as CDW diagrams with M instead of ρ. The magnetic flavor indices for each diagram can be read off from Fig. 5.

$$\tilde{M}^{[\ell]}_{k;\pm} = \left(\tilde{g}^{(\ell)M}_{k;00} - \tilde{g}^{(\ell)M}_{k;11} \pm \sqrt{(\tilde{g}^{(\ell)M}_{k;00} - \tilde{g}^{(\ell)M}_{k;11})^2 + 4\left|\tilde{g}^{(\ell)M}_{k;10}\right|^2}\right)\tilde{M}^{[\ell]}_{k;0} + 2\tilde{g}^{(\ell)M}_{k;10}\tilde{M}^{[\ell]}_{k;1}$$

and

$$\gamma^{[\ell]\rho}_{k;\pm} = \frac{1}{2}\left(\tilde{g}^{(\ell)\rho}_{k;00} + \tilde{g}^{(\ell)\rho}_{k;11} \pm \sqrt{(\tilde{g}^{(\ell)\rho}_{k;00} - \tilde{g}^{(\ell)\rho}_{k;11})^2 + 4\left|\tilde{g}^{(\ell)\rho}_{k;10}\right|^2}\right) \tag{S13}$$

$$\gamma^{[\ell]M}_{k;\pm} = \frac{1}{2}\left(\tilde{g}^{(\ell)M}_{k;00} + \tilde{g}^{(\ell)M}_{k;11} \pm \sqrt{(\tilde{g}^{(\ell)M}_{k;00} - \tilde{g}^{(\ell)M}_{k;11})^2 + 4\left|\tilde{g}^{(\ell)M}_{k;10}\right|^2}\right)$$

Supplementary Section II: Projected Gap Functions

Since the RG only determines the order parameter at the VHS points, it is necessary to extend it in some way to determine the nature of the resulting phase (chiral or nodal). In principle, one needs to extend the RG calculation to the whole BZ, which is computationally prohibitive already for moderate q. Even solving the self-consistent gap equation for a constant Hubbard interaction numerically is quite challenging. We therefore adopt a simpler approach and construct an ansatz gap function in real space in the $c_{\mathbf{r}\sigma}$ basis first (e.g. standard s- or d-wave gap functions with up to nearest neighbor terms, etc.), and then projecting onto the Hofstadter band of interest via $d_{\mathbf{k}\alpha\sigma} = \sum_s \mathcal{U}^s_\alpha(\mathbf{k})c_{\mathbf{k}s\alpha}$ with the band index α fixed.

The gap function at the VHSs is defined as

$$H_{SC,VHS} = \sum_{\ell,m,\mathrm{v}} \Delta^{(\ell)}_{m;\mathrm{v}} d^\dagger_{\ell+m,\mathrm{v}} d^\dagger_{-m,-\mathrm{v}} + h.c. \tag{S14}$$

where $\mathrm{v} = 0, 1$ is the VHS index and $m = 0, \ldots, q-1$ is the magnetic flavor index. The full gap function defined on the reduced MBZ is

$$\begin{aligned} H_{SC} &= \sum_{\ell,m,\mathbf{p}} \Delta_m^{(\ell)}(\mathbf{p}) d^\dagger_{\mathbf{p},\ell+m} d^\dagger_{-\mathbf{p},-m} + h.c. = \\ &= \sum_{m,n,\mathbf{p}} \hat{\Delta}_{mn}(\mathbf{p}) d^\dagger_{\mathbf{p},m} d^\dagger_{-\mathbf{p},n} + h.c \end{aligned} \tag{S15}$$

where the latter is the notation of [S1]. The gap function can be extended to include pairing between different bands α and β:

$$\begin{aligned} H_{SC} &= \sum_{m,n,\alpha,\beta,\mathbf{p}} \hat{\Delta}_{m\alpha,n\beta}(\mathbf{p}) d^\dagger_{\mathbf{p}\alpha m} d^\dagger_{-\mathbf{p}\beta n} + h.c = \\ &= \sum_{m,n,s,s',\mathbf{p}} \Delta_{m,s;n,s'}(\mathbf{p}) c^\dagger_{\mathbf{p}+m\mathbf{Q},s} c^\dagger_{-\mathbf{p}+n\mathbf{Q},s'} = \\ &= \sum_{\mathbf{R},\mathbf{R}',s,s'} \Delta_{\mathbf{R},s;\mathbf{R}',s'} c^\dagger_{\mathbf{R}s} c^\dagger_{\mathbf{R}',s'} \end{aligned} \tag{S16}$$

$\Delta_{m,s;n,s'}(\mathbf{p})$ is thus the gap function in the sub-lattice basis. We further define $\Delta^{(\ell)}_{ss'}(\mathbf{k})$ with $\mathbf{k}$ defined in the un-reduced MBZ via $\Delta_{m,s;n,s'}(\mathbf{p}) = \Delta^{(m+n)}_{ss'}(\mathbf{p} + (m-n)\mathbf{Q}/2)$. $\Delta^{(\ell)}_{ss'}(\mathbf{k})$ is then simply the gap function in the sub-lattice basis corresponding to pairing with total momentum $\ell\mathbf{Q}$ and defined on the original MBZ. We thus have

$$\begin{aligned} \hat{\Delta}_{m\alpha,n\beta}(\mathbf{p}) &= \sum_{ss'} \mathcal{U}^s_\alpha(\mathbf{p} + m\mathbf{Q}) \mathcal{U}^{s'}_\beta(-\mathbf{p} + n\mathbf{Q}) \Delta_{m,s;n,s'}(\mathbf{p}) \\ &= \sum_{ss'} \mathcal{U}^{s+m}_\alpha(\mathbf{p}) \mathcal{U}^{s'+n}_\beta(-\mathbf{p}) \Delta_{m,s;n,s'}(\mathbf{p}) \end{aligned} \tag{S17}$$

The second line follows from our gauge choice $\mathcal{U}^{s+1}_\alpha(\mathbf{k} + \mathbf{Q}) = \mathcal{U}^s_\alpha(\mathbf{k})$. We also have

$$\begin{aligned} \Delta_{m,s;n,s'}(\mathbf{p}) &= \frac{1}{N} \sum_{\mathbf{R},\mathbf{R}'} e^{-i(\mathbf{p}+m\mathbf{Q})\cdot\mathbf{r} - i(-\mathbf{p}+n\mathbf{Q})\cdot\mathbf{r}'} \Delta_{\mathbf{R},s;\mathbf{R}',s'} = \\ &= \frac{1}{N} \sum_{\mathbf{R},\mathbf{R}'} e^{-i\mathbf{p}\cdot\left(\mathbf{r}-\mathbf{r}'\right) - i\mathbf{Q}\cdot\left(m\mathbf{r}+n\mathbf{r}'\right)} \Delta_{\mathbf{R},s;\mathbf{R}',s'} \end{aligned} \tag{S18}$$

or equivalently

$$\begin{aligned} \Delta^{(\ell)}_{ss'}(\mathbf{k}) &= \frac{1}{N} \sum_{\mathbf{R},\mathbf{R}'} e^{-i\mathbf{k}\cdot\left(\mathbf{r}-\mathbf{r}'\right) - i\ell\mathbf{Q}\cdot\left(\mathbf{r}+\mathbf{r}'\right)/2} \Delta^{(\ell)}_{\mathbf{R},s;\mathbf{R}',s'} = \\ &= \frac{1}{N} \sum_{\mathbf{R},\mathbf{R}'} e^{-i\mathbf{k}\cdot\left(\mathbf{r}-\mathbf{r}'\right) - i\ell\mathbf{Q}\cdot\left(\mathbf{R}+\mathbf{R}'\right)/2} \Delta^{(\ell)}_{\mathbf{R},s;\mathbf{R}',s'} \end{aligned} \tag{S19}$$

Note that $\Delta^{(\ell)}_{\mathbf{R}+\mathbf{R}'',s;\mathbf{R}'+\mathbf{R}'',s'} = e^{i\ell\mathbf{Q}\cdot\mathbf{R}''} \bar{\Delta}^{(\ell)}_{\mathbf{R},s;\mathbf{R}',s'}$. The projection of $\Delta_{m,s;n,s'}(\mathbf{p})$ (or equivalently $\Delta_{\mathbf{R},s;\mathbf{R}',s'}$) onto the α band simply amounts to computing $\hat{\Delta}_{m\alpha,n\alpha}(\mathbf{p}) \equiv \hat{\Delta}_{mn}(\mathbf{p})$, assuming the rest of the components vanish.

We then seek $\bar{\Delta}_{\mathbf{R},s;\mathbf{R}',s'}$ such that $\hat{\Delta}_{mn}(\mathbf{K}_{0,v}) = \Delta^{(\ell)}_{m;v}$ as found in the RG calculation. Note that thanks to the MTG symmetry, we can look at the $\ell = m + n = 0$ channel alone, the rest being obtained by simple application of $\hat{T}_x$ symmetry (for $q > 2$ we then find the MTG symmetry of the ground state by minimizing the fourth order free energy in Supplementary Section III). In particular, for both $q = 2$ and the lower and upper bands for $q = 3$ we found $\Delta^{(0)}_{m;0} = -\Delta^{(0)}_{m;1}$. We also use the relations in this supplementary section to establish the action of the self-similarity symmetry $\hat{S}$ in different bases. Recall that it imposes $\Delta^{(0)}_{m;v} = -\Delta^{(0)}_{n;v}$ for all m and n (for $q = 2$ we find the same relation, but as a consequence of the usual $\hat{T}_x$ symmetry). On $\hat{\Delta}_{mn}(\mathbf{p})$, this symmetry acts as

$$\hat{\Delta}_{mn}(\mathbf{p}) \xrightarrow{\hat{S}} \hat{\Delta}_{m+1,n-1}(\mathbf{p}) = \left[\tau \hat{\Delta}(\mathbf{p}) \tau\right]_{mn} \tag{S20}$$

where $\tau_{mn} = \delta_{m,n-1}$ is the shift matrix. This is in contrast to the action of $\hat{T}_x$ itself, which acts as $\hat{\Delta}_{mn}(\mathbf{p}) \xrightarrow{\hat{T}_x} \left[\tau\hat{\Delta}(\mathbf{p})\tau^T\right]_{mn} = \hat{\Delta}_{m+1,n+1}(\mathbf{p})$. From the action of the $\hat{S}$ in the band basis, we establish its action on the gap function components in other bases: e.g., it acts on $\Delta^{(\ell)}_{ss'}(\mathbf{k})$ as

$$\Delta^{(\ell)}_{ss'}(\mathbf{k}) \xrightarrow{\hat{S}} \Delta^{(\ell)}_{s-1,s'+1}(\mathbf{k}+\mathbf{Q}) \tag{S21}$$

and as a convolution in real space:

$$\Delta_{\mathbf{R}s;\mathbf{R}'s'} \xrightarrow{\hat{S}} e^{-i\mathbf{Q}\cdot(\mathbf{R}-\mathbf{R}')} \sum_{X\in q\mathbb{Z}} \operatorname{sinc}\left[\frac{\pi}{q}(X+2)\right] \Delta_{\mathbf{R},s+1;\mathbf{R}'+X\hat{\mathbf{x}},s'-1} \tag{S22}$$

where $\operatorname{sinc}(x) = \sin x/x$ is the inverse Fourier transform of e^{2ik_x}.

Supplementary Section III: BdG Hamiltonian, Free Energy, and Edge Modes

In this section we discuss the Ginzburg-Landau free energy calculation used to establish the symmetry of the HSC for $q = 3$, as well as the edge mode calculation used for generating Fig. **??**. For both calculation we make use of the Bogoliubov-De Gennes (BdG) formalism. The starting point is the mean field Hamiltonian, which in the band basis reads

$$H = \sum_{\ell,\alpha,\mathbf{p}} \varepsilon_\alpha(\mathbf{p}) d^\dagger_{\mathbf{p},\ell,\alpha} d_{\mathbf{p},\ell,\alpha} + \frac{1}{2}\sum_{m,n,\alpha,\beta,\mathbf{p}} \left[\hat{\Delta}_{\ell,\alpha;\ell'\beta}(\mathbf{p}) d^\dagger_{\mathbf{p},\ell,\alpha} d^\dagger_{-\mathbf{p},\ell',\beta} + h.c.\right] + H_{\Delta^2} \tag{S23}$$

where $\alpha, \beta = 0, \ldots, q-1$ are the Hofstadter band indices, ℓ, ℓ' are the magnetic patch indices (we omit the spin index), and

$$H_{\Delta^2} = \sum_{\ell,n,m,\mathbf{p},\mathbf{p}'} \hat{\Delta}^\dagger_{\ell+m,-m}(\mathbf{p}) \left[g^{-1}(\mathbf{p};\mathbf{p}')\right]^{(\ell)}_{mn} \hat{\Delta}_{\ell+n,-n}(\mathbf{p}') \tag{S24}$$

is a term quadratic in the gap function arising from the Hubbard-Stratonovich transformation and involving the inverse of the coupling tensor:

$$\sum_{o\mathbf{q}} g^{(\ell)}_{mo}(\mathbf{p};\mathbf{q}) \left[g^{-1}(\mathbf{q};\mathbf{p}')\right]^{(\ell')}_{on} = \delta_{\ell,\ell'}\delta_{mn}\delta_{\mathbf{p}\mathbf{p}'} . \tag{S25}$$

Here we omitted the band indices in the interactions for simplicity as in the weak-coupling approximation we assume only interactions within a single band play a role and inter-band interaction will not play a role below (see previous Supplementary Section II). In the BdG formalism we introduce the Nambu spinors $\Psi_{\mathbf{p}\ell\alpha} = (d_{\mathbf{p}\ell\alpha}, d^\dagger_{-\mathbf{p}\ell\alpha})$, which allows us to write the Hamiltonian as

$$H = \frac{1}{2}\sum_{\ell,\ell',\mathbf{p}\alpha\beta} \Psi^\dagger_{\mathbf{p}\ell\alpha} \left[\mathcal{H}_{BdG}(\mathbf{p})\right]_{\ell,\alpha;\ell',\beta} \Psi_{\mathbf{p}\ell'\beta} + H_{\Delta^2} \tag{S26}$$

where

$$\left[\mathcal{H}_{BdG}(\mathbf{p})\right]_{\ell,\alpha;\ell',\beta} = \begin{pmatrix} \varepsilon_\alpha(\mathbf{p})\delta_{\alpha\beta} & \hat{\Delta}_{\ell,\alpha;\ell',\beta}(\mathbf{p}) \\ \hat{\Delta}^\dagger_{\ell,\alpha;\ell',\beta}(\mathbf{p}) & -\varepsilon_\alpha(-\mathbf{p})\delta_{\alpha\beta} \end{pmatrix} \tag{S27}$$

is the BdG Hamiltonian. Note that when $\hat{\Delta}_{\ell,\alpha;\ell',\beta} = 0$ for $\ell' \neq -\ell$ (i.e. when only zero total momentum pairing is present), one can remove the magnetic flavor indices ℓ and work on the non-reduced MBZ instead, replacing $\hat{\Delta}_{\ell,\alpha;-\ell,\beta}(\mathbf{p})$ with $\hat{\Delta}_{\alpha\beta}(\mathbf{p}+\ell\mathbf{Q})$, so that the BdG Hamiltonian is a $2q \times 2q$ matrix. In all other cases, however, the unit cell needs to be extended due to the breaking of the $\hat{T}_y$ MTG symmetry, resulting in the q-fold folding of the MBZ into the rMBZ, in which case we have to work with a $2q^2 \times 2q^2$ BdG Hamiltonian.

A. Ginzburg-Landau Free Energy

To obtain the gap function in the mean field approach we need to minimize the free energy, which yields the self-consistent gap equation. The free energy is in turn obtained from Eq. (S23) by integrating out the $\Psi_{\mathbf{p}\ell\alpha}$ fields from the partition function. For this part of the calculation we assume that the pairing happens only in one band α and so drop the band index. Using the Matsubara formalism we then find

$$\mathcal{F} = -T\sum_{\omega,\mathbf{p}}\mathrm{Tr}\left[\log\beta\mathcal{G}^{-1}(i\omega,\mathbf{p})\right] + H_{\Delta^2} \tag{S28}$$

where $\omega = (2\pi j+1)T$ with integer j are the Matsubara frequencies and we defined the Gor'kov Green's function

$$\begin{aligned}\mathcal{G}(i\omega,\mathbf{p}) &= (i\omega - \mathcal{H}_{BdG}(\mathbf{p}))^{-1} = \\ &= \begin{pmatrix} \hat{G}(i\omega,\mathbf{p}) & \hat{F}(i\omega,\mathbf{p}) \\ \hat{F}^\dagger(i\omega,\mathbf{p}) & -\hat{G}^T(-i\omega,-\mathbf{p})\end{pmatrix} .\end{aligned} \tag{S29}$$

Minimizing $\mathcal{F}$ with respect to $\hat{\Delta}^\dagger$ gives the gap equation

$$\hat{\Delta}_{\ell+n,-n}(\mathbf{p}) = T\sum_{\omega\mathbf{p}'m} g^{(\ell)}_{nm}(\mathbf{p};\mathbf{p}')\hat{F}_{\ell+m,-m}(i\omega,\mathbf{p}') . \tag{S30}$$

Close below T_c we can expand the free energy and the Green's functions in powers of the gap function and obtain the linearized gap equation (see the appendix in [S1] for details):

$$\hat{\Delta}_{\ell+n,-n}(\mathbf{p}) = -\log\frac{1.13\Lambda}{T}\sum_{\mathbf{p}'m}\nu(\mathbf{p}')g^{(\ell)}_{nm}(\mathbf{p};\mathbf{p}')\hat{\Delta}_{\ell+m,-m}(\mathbf{p}') \tag{S31}$$

where $\nu(\mathbf{p})$ is the momentum resolved density of states at the Fermi level and Λ is the high energy cutoff. We note that the linearized gap equation is equivalent to the 1 loop RG flow equation for the SC vertex and both give the same form of the gap function.

As shown in [S1] and discussed above, for odd q the gap function belongs to a q-dimensional irrep which means that just as in the 1 loop RG flow there are q degenerate solutions of the linearized gap equation that we can label $\hat{\Delta}^{(L)}$ with $L = 0,\ldots,q-1$ labeling the total momentum $L\mathbf{Q}$ of the corresponding Cooper pairs. The solutions are picked such that

$$\hat{\Delta}^{(L)} \xrightarrow{\hat{T}_x} \hat{\Delta}^{(L-2)} , \tag{S32}$$

$$\hat{\Delta}^{(L)} \xrightarrow{\hat{T}_y} \omega_q^{pL}\hat{\Delta}^{(L)} . \tag{S33}$$

$\hat{\Delta}^{(L)}$ then form q components of the irrep that are eigenmodes of the $\hat{T}_y$ symmetry and are generated by the $\hat{T}_x$ symmetry [S1]. Because the linearized gap equation is linear (as is the 1 loop RG vertex flow equation), any linear combination

$$\hat{\Delta}(\mathbf{p}) = \sum_L \eta_L\hat{\Delta}^{(L)}(\mathbf{p}) \tag{S34}$$

is a solution for any choice of the complex parameters η_L. The vector $\boldsymbol{\eta} = (\eta_0,\ldots,\eta_{q-1})$ constitutes the order parameter that is selected spontaneously once non-linear terms are included.

In order to obtain the higher order terms we take Eq. (S34) as the ansatz, plug it into the free energy in Eq. (S28) and sum over the momentum $\mathbf{p}$, which yields the macroscopic Ginzburg-Landau free energy

$$\mathcal{F} = a\left|\boldsymbol{\eta}\right|^2 + \sum_{MN} b_{MN}\sum_L \eta^*_{L+M}\eta^*_{L-M}\eta_{L+N}\eta_{L-N} \tag{S35}$$

where the parameters a and b_{MN} are

$$a = H_{\Delta^2} - \log\frac{1.13\Lambda}{T}\sum_{\mathbf{p}}\nu(\mathbf{p})\mathrm{Tr}\left[\hat{\Delta}^{(L)\dagger}\hat{\Delta}^{(L)}\right] , \qquad b_{MN} = \frac{7\zeta(3)}{32\pi^2T^2}\sum_{\mathbf{p}}\nu(\mathbf{p})\mathrm{Tr}\left[\hat{\Delta}^{(L+M)\dagger}\hat{\Delta}^{(L+N)}\hat{\Delta}^{(L-M)\dagger}\hat{\Delta}^{(L-N)}\right] . \tag{S36}$$

In order to compute the fourth order b_{MN} coefficients, we note that the point with high DOS $\nu(\mathbf{p})$ dominate in the sum, so we can get an approximate expression by restricting the sum only to the VHS points $\mathbf{K}_{\ell,\mathrm{v}}$. Minimizing the resulting Ginzburg-Landau free energy Eq. (S35) for the solutions we find in RG for $q=3$, we find that the solutions are of the form $|\eta_L| = \eta$, $\arg[\eta_L] = \pm 4\pi/3\delta_{LM}$ for some fixed $M = 0,1,2$, for a total of six solutions. As shown in [S1], these solutions are precisely the solutions symmetric under $\omega_3^{pM}\hat{T}_x\hat{T}_y^{\pm 1}$ for the $+$ and $-$ cases respectively (equivalently, the gap functions pick up a phase of ω_3^{-2pM} under $\hat{T}_x\hat{T}_y^{\pm 1}$). The fact that there are six solutions instead of three as one would expect from the breaking of the $\hat{T}_x$ symmetry is due to the fact that the $\hat{C}_4$ symmetry is also broken and maps $\hat{T}_x\hat{T}_y$ to $\hat{T}_y\hat{T}_x^{-1}$. In all cases the ground states have a $\mathbb{Z}_3$ symmetry.

B. Edge Mode Calculation

In order to compute the edge modes, we considered the BdG Hamiltonian for the gap function $\Delta_{ss'}(\mathbf{k})$ of the form in Eq. (12) put on a vertical cylinder, i.e. assuming a system periodic in the x direction but with open boundary conditions in the y direction. More precisely, we consider the $\hat{T}_x\hat{T}_y$ symmetric gap functions that are linear combinations $\sum_L \eta_L \Delta_{ss'}^{(L)}(\mathbf{k})$ satisfying $\Delta_{ss'}^{(L)}(\mathbf{k}) = \Delta_{s+1,s'+1}^{(L+2)}(\mathbf{k})$ with η_L found in the previous paragraph and with $\Delta_{ss'}^{(L)}(\mathbf{p}+\ell\mathbf{Q}) = \Delta_{L/2-\ell,s;L/2+\ell,s'}(\mathbf{p})$ as defined in Supplementary Section II. We then perform an inverse Fourier transform on k_y to obtain the total hybrid gap function

$$\Delta_{m,y,s;n,y',s'}(p_x) = \eta_{m+n}\Delta_0\left[\delta_{yy'}(1-\cos p_x) - \delta_{y,y'-1}\omega_3^{-p(s-s'+m-n)} + \delta_{y,y'+1}\omega_3^{p(s-s'+m-n)}\right] \tag{S37}$$

where for concreteness we took $\eta_L = \omega_3^{2\delta_{L,0}}$. The BdG Hamiltonian in this hybrid basis is then

$$[\mathcal{H}_{BdG}(p_x)]_{m,y,s;n,y',s'} = \begin{pmatrix} \mathcal{H}^{(0)}_{y,s;y',s'}(p_x)\delta_{mn} & \Delta_{m,y,s;n,y',s'}(p_x) \\ \Delta^*_{n,y',s';m,y,s}(p_x) & -\mathcal{H}^{(0)*}_{y,s;y',s'}(-p_x)\delta_{mn} \end{pmatrix} \tag{S38}$$

where

$$\mathcal{H}^{(0)}_{y,s;y',s'}(p_x) = \delta_{ss'}\left(-\mu\delta_{yy'} - 2t\delta_{y,y'-1}\omega_3^{ps} - 2t\delta_{y,y'+1}\omega_3^{-ps}\right) - t\delta_{yy'}\left(\delta_{s,s'+1}e^{ip_x} + \delta_{s,s'-1}e^{-ip_x}\right) \tag{S39}$$

is the normal state Hofstadter Hamiltonian in the hybrid basis. Taking the number of lattice sites along the y direction to be N_y, the hybrid BdG Hamiltonian is a $2q^2N_y \times 2q^2N_y$ Hamiltonian. In Fig. **??** we used $N_y = 100$ with $\mu = \pm 2.44$ and $\Delta_0 = 0.02$ and $\Delta_0 = 0.2$ respectively (setting $t=1$). We note that one could compute the Chern number directly from the bulk spectrum; however, interestingly we found that this numerical computation is more challenging than the edge mode computation.

[S1] D. Shaffer, J. Wang, and L. H. Santos, Phys. Rev. B **104**, 184501 (2021).
